# Prior Visual Experience Modulates Learning of Sound Localization Among Blind Individuals

**DOI:** 10.1007/s10548-017-0549-z

**Published:** 2017-02-04

**Authors:** Qian Tao, Chetwyn C. H. Chan, Yue-jia Luo, Jian-jun Li, Kin-hung Ting, Zhong-lin Lu, Susan Whitfield-Gabrieli, Jun Wang, Tatia M. C. Lee

**Affiliations:** 1 Psychology Department, School of Medicine, Jinan University, Guangzhou, China; 2 Applied Cognitive Neuroscience Laboratory, Department of Rehabilitation Sciences, The Hong Kong Polytechnic University, Hong Kong, Hong Kong; 3 National Key Laboratory of Cognitive Neuroscience and Learning, Beijing Normal University, Beijing, China; 4 China Rehabilitation Research Center, Beijing, China; 5 Center for Cognitive and Behavioral Brain Imaging, Arts, & Sciences, Department of Psychology, The Ohio State University, Ohio, OH 43210 USA; 6 The Gabrieli Lab, Massachusetts Institute of Technology, Cambridge, MA 02139 USA; 7 Laboratory of Neuropsychology, Department of Psychology, The University of Hong Kong, Hong Kong, Hong Kong; 8 Laboratory of Cognitive Affective Neuroscience, The University of Hong Kong, Hong Kong, Hong Kong; 9 State Key Laboratory of Brain and Cognitive Science, The University of Hong Kong, Hong Kong, Hong Kong

**Keywords:** Blindness, Experience modulation, fMRI, Functional connectivity, Sound localization

## Abstract

Cross-modal learning requires the use of information from different sensory modalities. This study investigated how the prior visual experience of late blind individuals could modulate neural processes associated with learning of sound localization. Learning was realized by standardized training on sound localization processing, and experience was investigated by comparing brain activations elicited from a sound localization task in individuals with (late blind, LB) and without (early blind, EB) prior visual experience. After the training, EB showed decreased activation in the precuneus, which was functionally connected to a limbic-multisensory network. In contrast, LB showed the increased activation of the precuneus. A subgroup of LB participants who demonstrated higher visuospatial working memory capabilities (LB-HVM) exhibited an enhanced precuneus-lingual gyrus network. This differential connectivity suggests that visuospatial working memory due to the prior visual experience gained via LB-HVM enhanced learning of sound localization. Active visuospatial navigation processes could have occurred in LB-HVM compared to the retrieval of previously bound information from long-term memory for EB. The precuneus appears to play a crucial role in learning of sound localization, disregarding prior visual experience. Prior visual experience, however, could enhance cross-modal learning by extending binding to the integration of unprocessed information, mediated by the cognitive functions that these experiences develop.

**Electronic supplementary material:**

The online version of this article (doi:10.1007/s10548-017-0549-z) contains supplementary material, which is available to authorized users.

## Introduction

Daily life requires the continuous integration of information obtained through multiple sensory modalities, which, in turn, depends on cross-modal learning and the use of information from various sensory modalities (Spence 2011). One example of cross-modal learning is visuoauditory spatial learning. This type of learning occurs when people use sensory-specific cues (i.e., visual and auditory) to infer multisensory representations characterizing intrinsic properties (i.e., locations) (Yamashita et al. 2015). For example, the contour of a train appears larger as the sound of its whistle becomes louder, both of which indicate that the train is arriving. The result of visuoauditory spatial learning enables individuals inside the lounge to realize that a train is arriving only by hearing its engine sound. Similar to other types of learning, cross-modal learning can be modulated by the experiences that individuals gain from the involved modalities. For example, individuals who have never heard the whistle would not recognize the sound but would associate a louder whistle with the arrival of the train.

Cross-modal learning is mediated by several neural networks that specialize in sensory association and memory (Calvert 2001). These networks consist of polymodal association regions, such as the medial parietal cortices, prefrontal cortices, and the superior temporal gyrus/sulcus (Watson et al. 2014), and memory-related regions, such as the hippocampus and parahippocampus (Tanabe et al. 2005). The specific network used largely depends on the sensory modalities involved and an individual’s familiarity with the sensory information being processed (Tanabe et al. 2005). Fuster et al. (2000) have proposed that cross-modal learning involves at least three steps: the activation of the cross-modal network during long-term memory formation; sustained activation of that association during working memory; and reactivation of the network when presented with one of the associates. Activity of the superior temporal sulcus was found to increase during the initial association process but to decrease as learning proceeded (Tanabe et al. 2005). Activity in the prefrontal cortex was related to the integration of visual and auditory stimuli that were sustained throughout the learning process (Fuster et al. 2000). This study investigated the effect of the prior experience of late blind individuals (LB) on modulating cross-modal learning. We are particularly interested in learning of sound localization, which involves comparisons of the associated processes between early blind (EB) and LB individuals.

There is vast literature on cross-modal learning in EB individuals based on the auditory (e.g., Chan et al. 2013; Halko et al. 2014; Striem-Amit et al. 2011) or tactile modality (Chebat et al. 2007, 2011; Kupers et al. 2010; Ptito and Kupers 2005). The common understanding among researchers is that EB individuals possess superior discrimination ability than do LB individuals, such as minimum-audible-angle discrimination in peripheral space (Voss et al. 2004) and high-resolution sound localization in a fan-shape space (Tao et al. 2015). In another two studies (Gougoux et al. 2005; Voss et al. 2011), which used the same/different-sound-position task and the pointing task toward sound sources, only those with superior performance among the EB participants were found to outperform their LB and normal-vision counterparts. The rest of the EB participants were reported to perform at a similar level to those of the LB or normal visual participants. Gori et al. (2014) tested congenitally blind participants on a spatial and temporal bisection task, a minimum audible angle task, a pointing to sound source task, and a slower version of the spatial bisection task. The results found that the participants, when compared with the sighted participants, only impaired at performing the spatial bisection task. In contrast, Kupers et al. (2010) trained congenitally blind participants on a virtual navigation task with a tactile-to-vision sensory substitution device. The participants showed significant improvements that were comparable to those of the sighted participants. In agreement with above results, two review studies presented both impaired and enhanced spatial skills of blind individuals across experimental tasks (Cuturi et al. 2016; Gori et al. 2016). A recent review study that Schinazi et al. (2016) conducted suggested that the differences observed could largely be due to the variations in the abilities, strategies taken, and mental representations of the blind individuals. More importantly, the study stipulated that blind individuals tended to show patterns of performances that could be progressively lower than (called cumulative), consistently lower than (persistent), or approaching (convergent) those of the sighted individuals (Schinazi et al. 2016). With this in mind, the task employed in this study made reference to the low-resolution sound recognition task used in our previous study on EB individuals who performed better than sighted participants did (Chan et al. 2013). The existing task was more challenging for the EB and perhaps LB, as it involved the localization of high-resolution sounds. Among the auditory and tactile modalities, this study chose to investigate sound localization learning for two reasons. First, the focus on the auditory modality extends our previous studies on sound localization processing (Chan et al. 2013; Tao et al. 2015) to further understand how prior visual experience would modulate its learning. Second, previous brain imaging studies reported common neural substrates mediating the cross auditory- and tactile-spatial processes (Renier et al. 2010). They clustered around the medial parietal areas, including the precuneus, superior parietal lobule (SPL), posterior parietal cortex (PPC), and middle occipital gyrus (MOG) (Bonino et al. 2008; Collignon et al. 2011; Renier et al. 2010). The results from the study of sound localization learning may shed light on the tactile-spatial counterpart in blind individuals.

Visual experience is important for the development of the multisensory integration necessary for spatial cognition (Pasqualotto and Proulx 2010). Such experience is more relevant to LB than EB individuals during the extraction of spatial information from the auditory signals. Neuroimaging studies explored the differences between EB and LB on cross sound localization processing. The results vary across studies but in general neural substrates in the occipital cortex particularly the MOG was consistently found to be activated only in EB but not LB group (Collignon et al. 2013; Tao et al. 2015; Voss et al. 2008). The MOG has been theorized to signify blind-related plasticity specialized in spatial localization of sound. Our previous study revealed that audio-spatial learning in EB participants was associated with activation of the inferior parietal cortex, left hippocampus, and right cuneus (Chan et al. 2013). It did not address how visual experience would have modulated the learning processes, and no LB participants were involved. The present study aimed to investigate the neural processes associated with cross-modal learning using a pre- and post-training design. The notion of visual experience was operationalized by comparing the EB and LB who had visual deprivation at the early and late developmental periods. The between-group comparison can also shed light on another issue related to learning ability of people blind individual. We proposed that prior visual experience in LB would facilitate sound localization learning and be associated with the increased activation of extended multi-modal association regions. Without prior visual experience, EB participants would gain less in the learning and have a lower increase in brain activations compared with LB participants. Prior visual experience was investigated by comparing EB and LB individuals on the sound localization learning of “Bat-ears” sounds through a 7-day training. This study did not have a normal vision control group because the results from a pilot study (N = 3) revealed that the training could yield below-random chance performances among the sighted individuals. More intense training, such as longer hours, for the normal vision participants would have biased the between-group comparison in the post-training scan. The “Bat-ears” is a sensory substitution device that utilizes an ultra-sound echo technology to assist blind individuals with navigation (Chan et al. 2013; Tao et al. 2015). The sounds emitted from the “Bat-ears” device contain spatial information with distance and azimuth. Longitudinal functional magnetic resonance imaging (fMRI) data were collected before and after the training while participants localized the “Bat-ears” sounds. Training-related changes in neural activation were investigated by region-of-interest (ROI) analysis and longitudinal contrast analysis; training-related changes in functional connectivity were investigated by the psychophysiological interaction (PPI) analysis (Friston et al. 1997). The PPI method has been shown to be effective for studying integrative processes across sensory modalities (Kim and Zatorre 2011). The hypothesis is that prior visual and multisensory spatial experience in LB would facilitate sound localization learning through visuo-spatial working memory. The LB participants with high visuo-spatial working memory abilities would improve more than LB participants with low visuo-spatial working memory abilities. Sound localization learning of better learners would be associated with increased activation of the polymodal association regions. Without prior visual experience, EB participants would only be able to learn the pairing of the sound localization stimuli and it would be associated predominantly with the memory network. Because of the differences in the postulated learning processes, we further hypothesized that EB participants would not have superior level of performance than the LB participants.

## Materials and Methods

### Participants

There were 14 EB and 17 LB participants initially recruited, who participated in a previous cross-modal processing study reported in Tao et al. (2015). Three EB and 4 LB participants dropped out of the study during training due to personal reasons unrelated to the training. The final sample size was 11 EB (6 male, age range = 19–31 y, mean age = 26.36) and 13 LB (13 male, age range 27–49 y, mean age = 33.85 y) participants. The EB participants were affected by congenital blindness that presented before the first year of age. In the LB group, the onset of blindness ranged from 5 to 39 years (mean = 21.23 y) while the duration of blindness ranged from 2 to 31 years (mean = 12.62 y). The demographics of the participants are summarized in Table 1. All participants reported no light perception and normal hearing. To ensure that all participants were able to discriminate auditory stimuli, we used a pitch discrimination test with a criterion of above 60% accuracy (Collignon et al. 2007). The stimulus pairs composed of a reference sound (200 ms) and a discrimination sound (200 ms). The test was a one-back comparison task, which required the participants to compare and determine whether the two stimuli were same or different regarding its identity (frequency or intensity). The EB (mean = 77.8%) and LB participants (mean = 75.5%) demonstrated comparable discrimination performance (t_2,22_ = 0.64, *P* = 0.529). Furthermore, all participants had normal intelligence, as assessed by the Wechsler Adult Intelligence Scale-Revised for China (WAIS-RC) (Gong 1982; Wechsler 1955). A pilot study was conducted on three normal vision individuals (2 male, age range = 25–29 years, mean age = 27.33) and they were blindfolded throughout testing and training. The mean accuracy performance on the pitch discrimination test was 62.3% and the sound localization performance was improved from 23.4 to 29.7% (below the chance level of 33.3%). It suggested that the sound localization task (15 sounds) constructed for this study had been too difficult to learn and perform by normal vision individuals. As a result, only EB and LB individuals were recruited as participants in this study. Written informed consents were obtained from all participants and they understood the purpose and procedure of the study. The research protocol was approved by the Human Ethics Committees of Beijing Normal University, where the fMRI scans were carried out, and The Hong Kong Polytechnic University, from which the study originated. The methods were carried out in accordance with the approved protocols/guidelines.

**Table 1:** Demographic characteristics of the participants

Subject number	Education	Gender	Age	Etiology	Onset of blindness
EB group (n = 11)					
1	Vocational education	M	22	Congenital cataract	Birth
2	Vocational education	M	20	Congenital glaucoma	Birth
3	Secondary school	F	29	Retintis pigmentosa	1 year
4	High school	F	31	Retintis pigmentosa	Birth
5	Vocational education	M	28	Congenital cataract	Birth
6	High school	F	19	Congenital optic atrophy	Birth
7	Vocational education	F	26	Congenital optic atrophy	Birth
8	Vocational education	M	31	Congenital cataract	<1 year
9	Vocational education	M	28	Optic nerve damage	Birth
10	Vocational education	F	30	Retintis pigmentosa	Birth
11	Secondary school	M	26	Congenital optic atrophy	Birth
LB group (n = 13)					
1	High school	M	38	Retinal datachment	36
2	Secondary school	M	49	Optic nerve damage	39
3	High school	M	28	Retinal datachment	15
4	High school	M	28	Retintis pigmentosa	10
5	Vocational education	M	45	Retintis pigmentosa	35
6	High school	M	32	Retinal datachment	22
7	Secondary school	M	35	Retinal datachment	26
8	High school	M	32	Optic nerve damage	30
9	Vocational education	M	29	Congenital glaucoma	5
10	Vocational education	M	30	Ocular fundus disease	18
11	Secondary school	M	38	Ocular fundus disease	7
12	Secondary school	M	27	Cataract	15
13	Vocational education	M	29	Congenital glaucoma	18

### Behavioral Test–Matrix Test

The adapted matrix test (Cornoldi et al. 1991; Tao et al. 2015) was used to assess visuo-spatial working memory ability among the participants. There were two haptic subtests: one 2D matrix (3 × 3 squares) comprised of nine wooden cubes (2 cm per side) and one 3D matrix (2 × 2 × 2 squares) comprised of 8 wooden cubes (2 cm per side). Within each matrix, sandpaper pads were attached to the surface of one cube, which was easily tactually recognized as a target. For each trial, the starting position of the target was varied and the participant was instructed to tactually recognize and memorize the location of the target. The participant was then instructed to mentally maneuver the target on the surface of the matrix according to verbal scripts, which were delivered to the participant using a tape recorder. The verbal scripts were instructions on relocating the target, such as forward–backward and right-left for the 2D matrix or forward–backward, right-left and up-down for the 3D matrix. Finally, the participant was required to indicate the final location of the target on a blank 2D or 3D matrix. The task requires active visuo-spatial imagery operations on matrices and tests for short-term memory of visuo-spatial materials. The task progressed from easy to difficult trials. For a difficult trial, two or three targets were maneuvered in two to four steps of relocation instructions. There were 12 trials each for the 2D and 3D matrices. Performance was measured as the percentage of final locations accurately identified out of the 24 trials by the participant.

### Stimuli and Apparatus

**Fig. 1:**
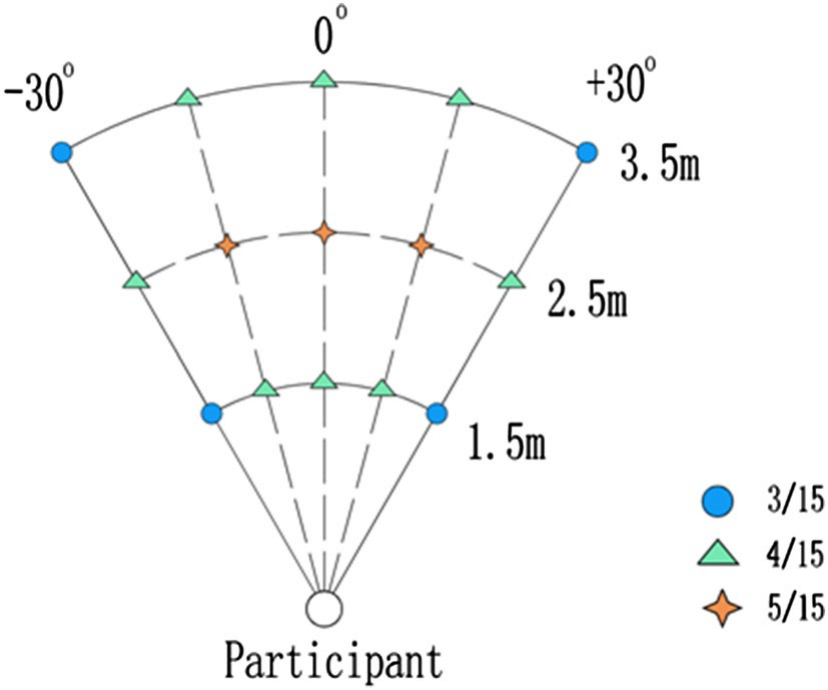
Locations for the low- and high-resolution sound stimuli and definition of correct responses in the sound localization task. The neighboring locations are also regarded as correct responses. The neighboring locations have the same distance or azimuth as the exact correct locations, but with one-step difference in distance or azimuth. The locations indicated as *blue circles* (e.g.,1.5 m/−30°), each has two neighboring locations; the locations indicated as *green triangles* (e.g., 2.5 m/−30° or 1.5 m/−15°), each has three neighboring locations; the locations indicated as *orange stars* (e.g., 2.5 m/+15°), each has four neighboring locations. The chance levels of accuracy for the *blue, green* and *orange* locations are 33.33, 25, and 20%, respectively. (Color figure online)

The auditory stimuli originated from an auditory substitution device called “Bat-ears.” The electronic “Bat-ears” device was used in two other studies conducted by the authors and described elsewhere in detail (Chan et al. 2013; Tao et al. 2015). In brief, it consists of one transmitter, two receivers, one demodulator, and two earphones. The transmitter emits ultrasonic pulses, which are reflected back as echoes upon hitting an obstacle. The binaural receivers then detect the reflected echoes, which are converted to audible signals (*da*-*da*-*da* sounds) by the demodulator. Finally, audible sounds are received by human subjects through the earphones. The KEMAR Manikin (Burkhard and Sachs 1975) was used to record the “Bat-ears” stimuli that reflected from the obstacle (30 × 30 cm cardboard), which was placed at designated locations in a soundproof chamber. Low-resolution stimuli were recorded at six locations [two distances: 1, 4 m; paired with 3 azimuths: −30° (left side), 0°, and +30° (right side)], and the high-resolution stimuli were recorded at 15 locations [3 distances: 1.5, 2.5, 3.5 m; paired with 5 azimuths: −30° (left side), −15°, 0°, +15°, +30° (right side)] (Fig. 1). The auditory stimuli were bilaterally presented via MRI-compatible headphones, and the sound-pressure level was adjusted to 80–90 dB.

### Sound Localization Training

The sound localization training proceeded from low- (6 locations) to high-resolution (15 locations) stimuli, with 2 and 5 sessions (2 h per session), respectively. The training was conducted in the sitting position. Verbal instruction describing the sound-location pair was given prior to listening to the sound. A 2D fan-shaped model was constructed and used to facilitate the communication between the participant and the trainer. The locations were further explained with the trainer by guiding the participant’s hands to the corresponding physical locations on the 2D model. The training was divided into distance and azimuth training blocks (Bedford 1993, 1995). The sequence of the two types of training blocks was counterbalanced. An evaluation trial was conducted for each of the distance and azimuth training block, in which the participant received verbal feedback on their performance. The participant had to reach 80% accuracy in both distance and azimuth training blocks, after which the participant could proceed to the final training stage for localization evaluation (both distance and azimuth). Two reasons exist for setting an 80% accuracy rate. First, the sound localization task is relatively hard for both EB and LB participants to perform. To maintain a low attrition rate, the 80% is to enable the participants to complete the training without spending an excessively long amount of time on it. This also helps to minimize the frustrated experiences of the participants. Second, the 80% is to safeguard an adequate number of correct trials for the fMRI analysis and hence reach a good signal-to-noise ratio. At the end of training, all participants obtained a performance level of 60% accuracy on the high resolution sound localization. The mean number of blocks used for evaluation is six (range from 5 to 8) and it is same for EB and LB. As a result, the training blocks only included verbal instruction by the trainer and did not have performance. The evaluation trials using a correction feedback procedure had performance, and both EB and LB had to achieve performance of 80% for each distance and azimuth block and obtain performance of 80% for localization (distance plus azimuth).

### Experimental Design and fMRI Tasks

fMRIs were conducted pre- and post-training. An event-related design was used. The participants went through a familiarization session (1 h) before the pre-training scan, which involved practicing on six sound-to-location trials using a headphone and joystick. Each scan session had four functional runs. Each run had an unbalanced number of localization (experimental, 17–20) and discrimination (control, 8–11) trials organized in a pseudo-randomized order. The order of the runs was counterbalanced among the participants. This gave a total of 75 localization and 37 discrimination trials. For each trial, an auditory cue (750 ms) was presented to indicate the task type: either localization (2000 Hz, 70 dB) or discrimination (500 Hz, 70 dB). Following the tone presentation, there was a 1750 ms delay during which the participant was instructed to prepare for the appropriate task. The “Bat-ears” stimulus was presented for 3000 ms followed by a 500 ms auditory cue (2000 Hz, 70 dB) after which the participant was to respond with the joystick. The response window was 4000 ms. Successive trials were separated with a jitter sampled from a uniform distribution (2500, 5000, or 7500 ms). The sound localization task required the participant to hear the “Bat-ears” sounds and identify their locations among the 15 combinations of azimuth and distance. The participant responded by maneuvering the joystick to one particular location, which indicated both the distance and azimuth of the sound source. Calibration of the joystick was: left/straight/right indicated by −15°/0°/+15° and outer left/right indicated by −30°/+30°; and backward/horizontal/forward indicated by 1.5/2.5/3.5 m. The pitch discrimination task was designed as a control task and required the participants to discriminate whether a deviant pitch (6000–8000 Hz, 70 dB) was inserted into the “Bat-ears” sounds. The participant made a “Yes” or “No” response by pressing or by not pressing on the joystick, respectively. In both tasks, we used novel “Bat-ears” sounds, which were recorded near the locations of the high-resolution sounds used in the training. This discrimination task was meant to produce baseline BOLD responses associated with non-auditory spatial processing.

### Behavioral Analysis

Response time for the localization task was not used as a behavioral measure because responses to stimuli presented at farther distances (e.g., 3.54 m) and at the outer left or right side (e.g., ±31°) took longer to register responses to than did those at a closer distance (e.g., 1.47 m) and at the center (e.g., ±2°). Most participants reported that localizing the sounds and mapping the location on joystick required some effort particularly when the task was carried out in the scanner. As a result, more lenient criteria were adopted for defining accuracy of the responses (Fig. 1): localization of an exact or neighboring location was regarded as a correct response. For instance, responses at two neighboring locations were regarded as “correct” for localizing a stimulus emitted from the nearest-distance outer-right location (1.53 m, +30°). The two neighboring locations were the medium-distance outer right (2.46 m, +30°) and nearest-distance right (1.52 m, +13°). Accuracy rate was defined as the percentage of correct trials over all the trials completed by the participant during each of the two scans. The effect of training on the accuracy rate were tested using repeated measure ANOVA: Group (LB versus EB) × Training (Pre-training versus Post-training). Significant interaction effects were tested with post-hoc comparisons. All tests adopted *P* < 0.05 as the threshold for defining statistical significance.

### MRI Acquisition and Analysis

The method of data acquisition was comparable to that used in Tao et al. (2015). fMRI series were acquired on a 3-T Siemens machine with a 12-channel head coil. Functional T2^*^-weighted images were obtained with a gradient echo-planar sequence (repetition time [TR] = 2500 ms; echo time [TE] = 30 ms; flip angle [FA] = 90°; voxel size = 3.1 × 3.1 × 3.2 mm^3^). Structural T1-weighted images (TR = 2530 ms; TE = 3.39 ms; voxel size = 1.3 × 1.0 × 1.3 mm^3^) were also acquired.

Analyses were carried out using SPM8 (Welcome Department of Imaging Neuroscience, London, UK) implemented in MATLAB R2008a (Mathworks). Preprocessing included slice-timing correction to correct for differences in the timing of acquisition between slices, realignment of functional time series to remove head motion, co-registration of functional and anatomical data, segmentation for extracting gray matter, spatial normalization to the Montreal Neurological Institute (MNI) space, and spatial smoothing (Gaussian kernel, 6 mm FWHM).

The preprocessed fMRI data were fitted to a general linear model (GLM) in SPM8 (Friston et al. 1994). We used four event-related regressors. Two of those modeled the BOLD signals corresponding to the 3000 ms “Bat-ears” sounds in correct responses made in the localization and discrimination trials. The other two were regressors of no interest that modeled the BOLD signals for incorrect responses made in the localization and discrimination trials. All regressors were constructed by convolving the onset times of the “Bat-ears” sounds with the canonical hemodynamic response function. The motion parameters detected by the Artifact Detection Tools (ART, developed by the Gabrieli Lab, Massachusetts Institute of Technology, available at: http://web.mit.edu/swg/software.htm) were included in the GLM for further regression of the motion-dependent confounds (Mazaika et al. 2005). Slow changes in the data were removed by applying a high-pass filter with a cut-off of 128 s, and a first-order autoregressive process was used to correct for autocorrelation of residuals in the GLM. Linear contrast of (Localization–Discrimination) was used to test the main effect of interest: sound localization processing. After single-subject analyses, we performed random-effect analyses at the group level for the EB and LB groups on the pre- and post-training sessions. The results of the pre-training session partially overlapped with those reported in Tao et al. (2015). The reason for the partial overlap was that not all participants in the pre-training completed the training. One-sample *t* tests were performed to investigate the main effect of sound localization. In addition, we conducted the following contrasts at individual level to investigate the training effect: (Localization_pre_ − Discrimination_pre_) − (Localization_post_ − Discrimination_post_) and (Localization_post_ − Discrimination_post_) − (Localization_pre_ − Discrimimation_pre_). The subtraction at the individual level was to control for the effects of repeated measurement and time (Poldrack 2000; Poldrack and Gabrieli 2001). One-sample *t* test was conducted in the EB and LB groups to characterize the training effect.

To address how visual experience could modulate sound localization training, exploratory ROI analyses were performed on the basis of the current results. The ROIs were defined by conjunction analysis (Nichols et al. 2005) of the pre- and post-training session data. The conjunction analysis identified common BOLD responses across all participants. The threshold was *P* < 0.001 (uncorrected) at the voxel level and *P* < 0.05 (FDR corrected) at the cluster level. All ROIs were created with a spherical mask 9 mm in radius centered at the local peaks of the activated clusters. Then, a stand-alone MATLAB-based toolkit of REX was used to extract the mean contrast value of the ROIs, which was submitted to 3 (Group: EB versus LB-LVM versus LB-HVM) × 2 (Training: Pre- vs Post-training) repeated measure ANOVA. To assess correlation with behavioral performance, Pearson correlation analyses were conducted on changes of mean contrast value of ROIs and performance in the sound localization task.

Finally, to further explore the training-dependent changes on functional connectivity, PPI analyses (Friston et al. 1997) were performed. As with the ROIs in the ROI analysis, seed regions were defined by conjunction analysis (Nichols et al. 2005) of the pre- and post-training session data. Separate PPIs were computed for the EB, LB-LVM and LB-HVM groups. In each participant, BOLD signal time series were extracted from the seed regions (6 mm sphere) and entered into the PPI analysis. New GLMs were constructed with four regressors: (i) the psychological regressor representing the main effect of auditory spatial processing (Localization–Discrimination), (ii) the physical regressor representing the original VOI eigenvariate in each session, (iii) the interaction of interest between the psychological and physical regressors, and (iv) the movement regressor. The 1st level GLM was built on pre- and post-training sessions and each session therefore has four regressors (psychological, physiological, interaction, motion). Significant PPI results indicated that the reported regions were functionally connected with the seed region in response to auditory spatial processing. The individual summary images (fixed effects) were then spatially smoothed (6 mm FWHM Gaussian kernel) and entered in group-level one-sample *t* test to test the auditory spatial processing effect for each session in each group (random effects). The threshold was *P* < 0.005 (uncorrected) at the voxel level and *P* < 0.05 (FDR-corrected) at the cluster level.

## Results

### Behavioral Results–Matrix Test

The 2D and 3D matrix test were administered to test the visuo-spatial working memory of the participants. The results of independent sample *t*-test showed significant better performance on the 2D test in the LB (mean = 58.2 ± 15.3%, ranged 40.0–86.7%) than the EB group (mean = 43.9% ± 12.7, ranged 33.3–70%) (t_2,22_ = 2.46, *P* = 0.022). For the 3D test, the LB group (mean = 49.2 ± 20.2%, ranged 26.7–90.0%) performed not significantly differed from the EB group (mean = 37.3 ± 14.3%, ranged 26.7–66.7%) (t_2,22_ = 1.64, *P* = 0.114). Given the large variance and potential heterogeneity in the LB group, we further differentiate the LB participants according to their performance on 2D test. A cut-off score of 50% for the 2D test was set for classifying the LB participants into the higher or lower ability groups. The participants with aggregated accuracy rates higher than 50% formed the higher VM group (LB-HVM; n = 7), whereas those having rates equal to or lower than 50% formed the lower VM group (LB-LVM; n = 6).

### Behavioral Results–fMRI Task

**Fig. 2:**
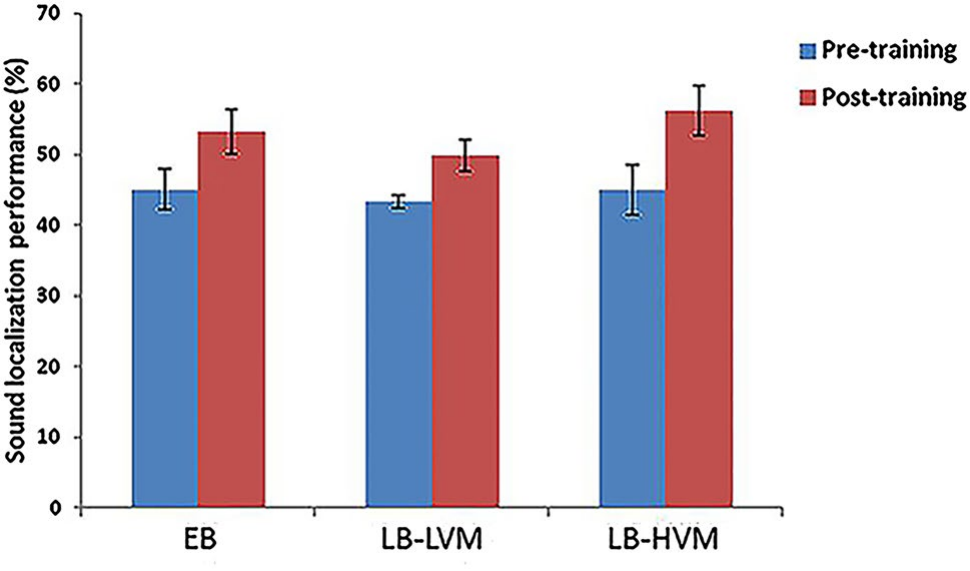
Behavioral performance on the sound localization task in the EB (n = 11), LB-LVM (n = 6), and LB-HVM (n = 7) groups

For the sound localization trials, all participants performed above the chance level of 33.3% at both pre- and post-training occasions. The first round of analysis was based on the two groups of LB (n = 13) and EB (n = 11) participants. A significant Training effect was revealed (*P* < 0.001), and both the EB (Pre-training: mean = 45.09 ± 5.62%; Post-training: mean = 53.21 ± 6.41%) and the LB (Pre-training: mean = 44.21 ± 5.22%; Post-training: mean = 53.23 ± 6.65%) groups showed a higher level of performance after the training. No significant Group (F_1,22_ = 0.034, *P* = 0.856) and Group × Training effects were revealed (F_1,22_ = 0.404, *P* = 0.531). The second round of analysis was based on the three groups of LB-LVM (n = 7), LB-HVM (n = 6), and EB (n = 11) participants. In contrast to the first round of analysis, a significant Group × Training effect was revealed (F_2,21_ = 4.35, *P* = 0.026), suggesting that participants in the three groups (EB, LB-HVM, and LB-LVM) benefited differently from the sound localization training (Fig. 2). Subsequent post-hoc analyses were based on the change in performance score. As expected, members of the LB-HVM group learned sound localization significantly better (Pre-training: mean = 44.95 ± 7.04%; Post-training: mean = 56.19 ± 7.03%) than members of the LB-LVM (*P* = 0.046; Pre-training: mean = 43.33 ± 2.02%; Post-training: mean = 49.78 ± 4.52%) and EB (*P* = 0.042; Pre-training: mean = 45.09 ± 5.62%; Post-training: mean = 53.21 ± 6.41%) groups. No significant differences were revealed between the LB-LVM and EB groups.

For the discrimination trials, the EB (Pre-training: mean = 79.61 ± 10.63%; Post-training: mean = 81.57 ± 10.17%) and the LB group (Pre-training: mean = 79.58 ± 9.41%; Post-training: mean = 79.79 ± 11.72%) had comparable performances before and after the training. No significant Training (F_1,22_ = 0.21, *P* = 0.651), Group (F_1,22_ = 0.063, *P* = 0.804), or their interactions (F_1,22_ = 0.139, *P* = 0.713) were revealed.

### Sound Localization Learning–Training Effect

**Fig. 3:**
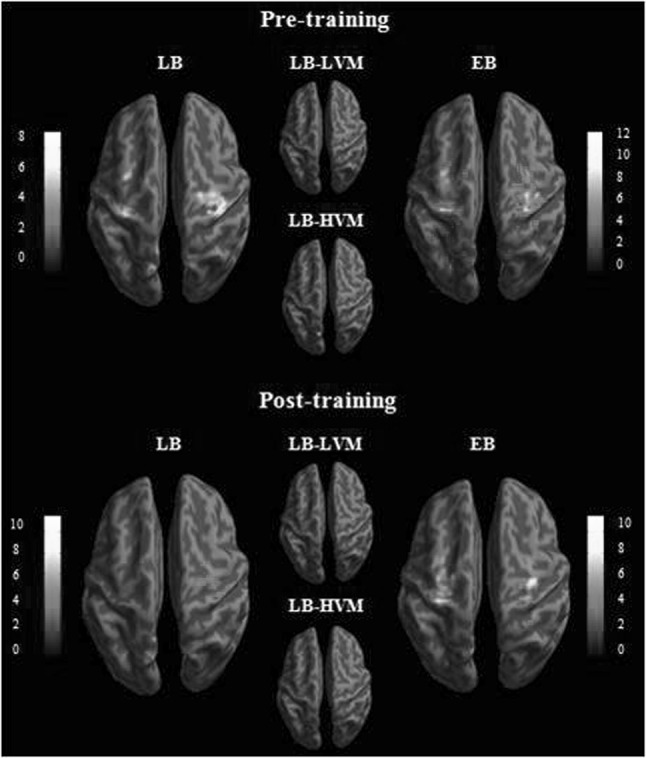
*t* maps for contrasts testing the main effect of sound localization processing (Localization–Discrimination) on the pre-training and post-training scan. *P* < 0.001 at the voxel level

**Fig. 4:**
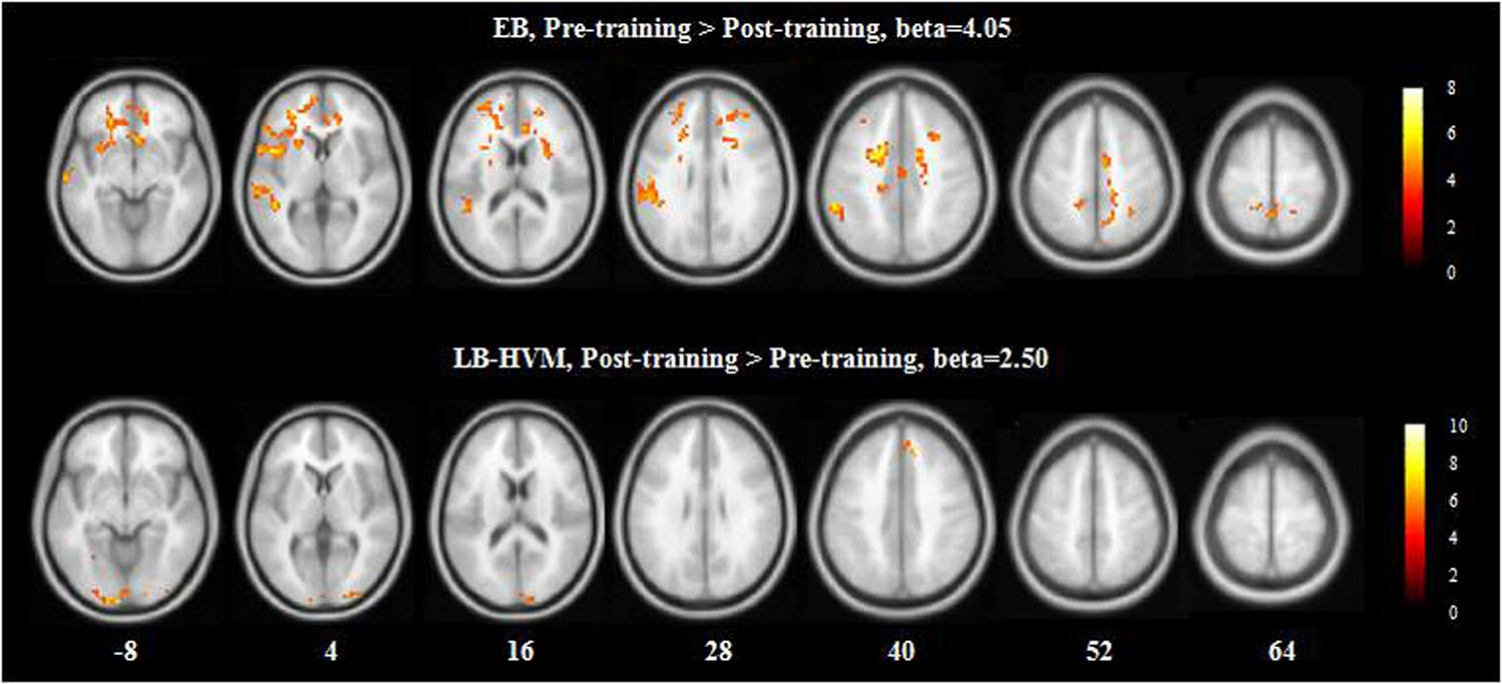
*t* maps for longitudinal contrasts testing the modulation effect of the training. *P* < 0.005 at the voxel level

The results of *t*-tests for the contrast of (Localization–Discrimination) in the EB and LB groups (including the two subgroups) during the two scanning sessions are shown in Fig. 3 (*P* < 0.001 uncorrected). As expected, both EB and LB groups showed significant BOLD responses in the bilateral occipital regions and parieto-frontal clusters, including the precentral gyrus, postcentral gyrus, and precuneus (Supplemental Table 1). However, longitudinal contrast analyses did not reveal significant results in the LB. Further analyses revealed different post- versus pre-training patterns of change in the BOLD responses across the EB and LB-HVM groups (Fig. 4, *P* < 0.005 uncorrected). The LB-HVM group showed increased BOLD responses in the left lingual gyrus, whereas the EB group showed decreased BOLD responses in the left postcentral gyrus, left cingulate gyrus, left inferior temporal gyrus (ITG), right precuneus, and right middle temporal gyrus (MTG) (Table 2).

**Table 2:** Longitudinal analyses results for the training effect in the EB, LB-LVM, and LB-HVM groups

Label	Cluster size	x (mm)	y (mm)	z (mm)	T
Training induced increase in EB					
NA					
Training induced decrease in EB					
L Postcentral gyrus	419	−56	−25	24	9.37*
L Inferior temporal gyrus	95	−47	−41	−15	8.13*
L Cingulate gyrus	1531	−16	6	43	8.07*
L Middle occipital gyrus	46	−28	−72	14	7.94
R Precuneus	253	15	−47	59	7.32*
R Middle temporal gyrus	211	49	−44	−12	6.90*
R Sub-Gyral	45	34	−75	8	6.50
Training induced increase in LB-HVM					
L Cuneus	38	21	−97	−2	11.78
R Cerebellum	16	24	−81	−40	11.44
L Lingual gyrus	65	−7	−100	−8	7.58*
L Fusiform gyrus	16	−35	−56	−18	7.19
R Medial frontal gyrus	16	9	37	40	6.96
R Cuneus	18	15	−100	14	6.67
R Precuneus	10	9	−62	46	6.05
Training induced decrease in LB-HVM					
NA					
Training induced increase in LB-LVM					
NA					
Training induced decrease in LB-LVM					
NA					

*P* < 0.005 (uncorrected) at the voxel level

*Significant at *P* < 0.05 (FDR corrected) at the cluster level. *L* left, *R* right

### Sound Localization Learning–Experience Modulation

To test whether the modulation of the sound localization training would be differed in the two groups, two sample *t*-tests were performed on the longitudinal contrasts. The modulation effects for the contrast of (Localization_pre_ − Discrimination_pre_) − (Localization_post_ − Discrimination_post_) was significantly more intense in the EB than the LB-HVM in the left inferior frontal gyrus (IFG) and left superior temporal gyrus (STG). In contrast, the modulation effects for the contrast of (Localization_post_ − Discrimination_post_) − (Localization_pre_ − Discrimination_pre_) was significantly more intense in the LB-HVM than the EB in the left medial frontal gyrus (MeFG) and left STG.

**Fig. 5:**
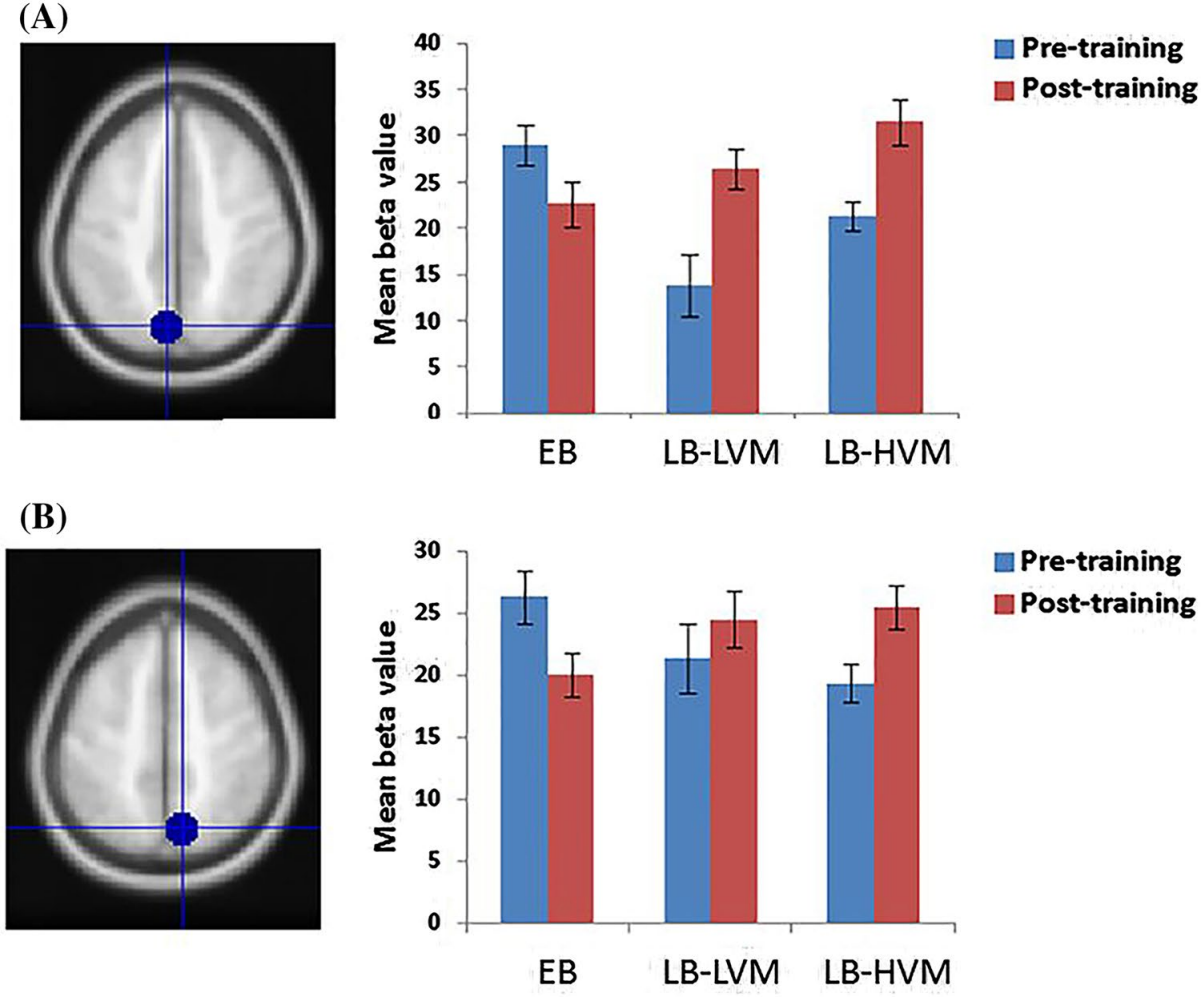
ROI results showed a significant Group × Training interaction effect. **a** The left precuneus. **b** The right precuneus

The ROIs were defined from conjunction analyses on BOLD responses before and after sound localization training. Four ROIs were identified: the right precuneus (9, −69, 52), the right MeFG (6,3,52), the left precuneus (−7, −72, 46), and the left MFG (−28, −0,65). Repeated ANOVA based on the three-group model revealed a significant Group × Training effect on BOLD signal changes in the left precuneus (F_2,21_ = 3.75, *P* = 0.041) and a marginally significant effect in the right precuneus (F_2,21_ = 3.04, *P* = 0.069, *ƞ* ^*2*^ = 0.224). Opposite changes in contrast value in the two neural correlates were revealed between the LB and EB groups, with increased change in contrast value in both the LB-HVM and LB-LVM groups but decreased change in the EB group (Fig. 5). Furthermore, sound localization performance in both the LB-HVM and LB-LVM groups was moderately correlated with the increased activation of the right precuneus (r = 0.589, *P* = 0.034) and the left MFG (r = 0.599, *P* = 0.031) as well as a marginal significant correlation with increase in the left precuneus (r = 0.504, *P* = 0.079), whereas no significant correlation with increase was observed in the right MeFG (r = 0.259, *P* = 0.393).

**Fig. 6:**
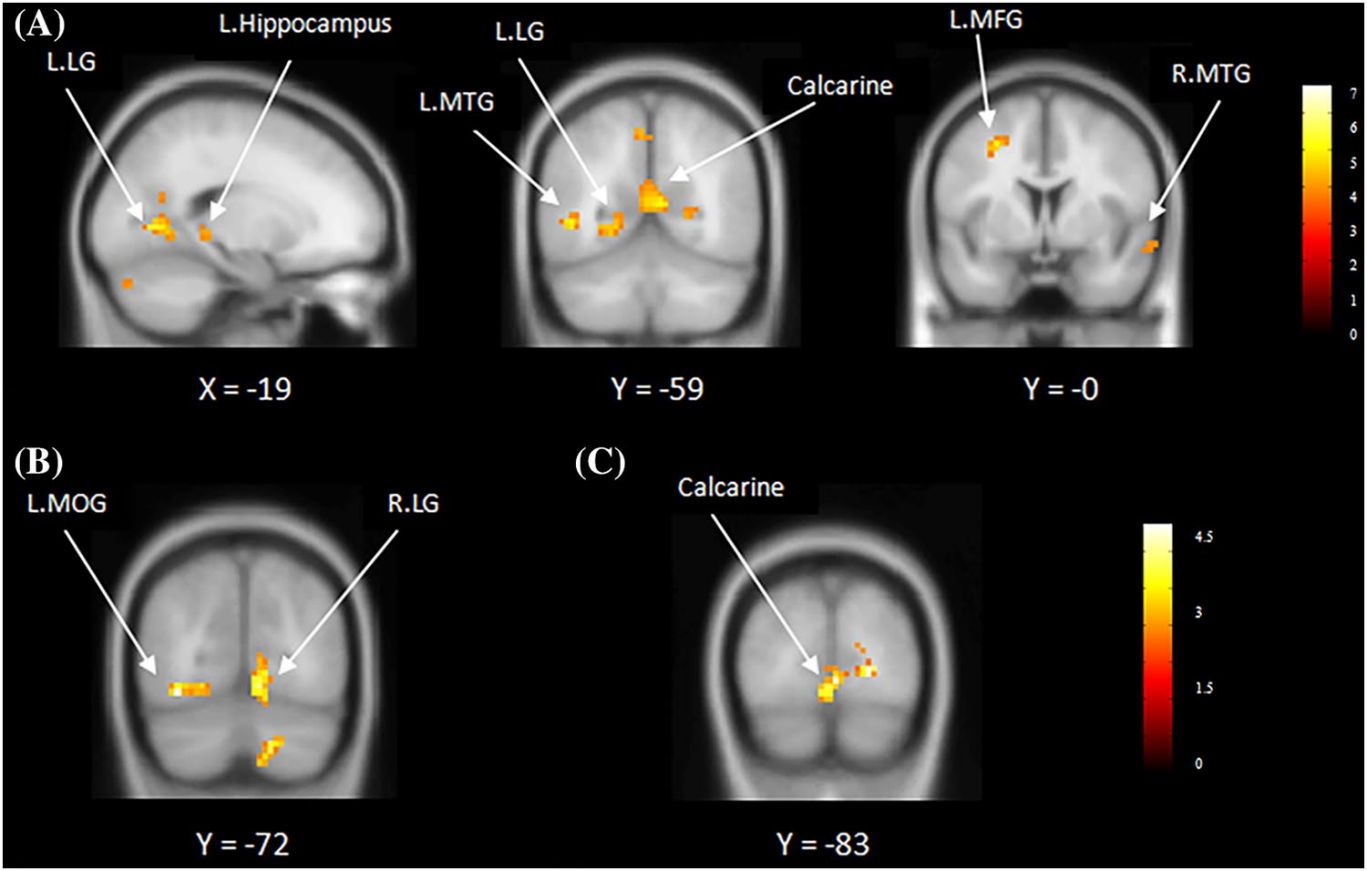
PPI results showed training enhanced functional connectivity between **a** the left precuneus and a distributed network in the EB group, **b** the left precuneus and occipital regions in the LB-HVM group, and **c** the right precuneus and occipital regions in the LB-HVM group

The four ROIs identified above were used as seed regions for conducting the PPI analyses. For the LB-HVM group, enhanced connectivity was revealed between the right precuneus and left lingual gyrus and between the left precuneus and the right lingual gyrus; no significant enhancement of connectivity was found in the LB-LVM group. For the EB group, the left precuneus was significantly connected to extensive limbic-temporo-parietal networks in the left and right brain. The networks included the left hippocampus, right posterior cingulate gyrus, bilateral MTG, right STG, precuneus, and inferior parietal lobule (IPL) (Fig. 6) (Supplemental Table 2).

### Subgroup Analysis for EB and Total Participants

The modulation effect of visuo-spatial working memory on sound localization learning was further tested among the EB participants. The analysis procedures used for the LB participants were replicated. The cut-off score of 40% on the 2D test was used to classify the EB participants into higher and lower ability subgroups. Those who obtained an aggregated accuracy rate equal to or higher than 40% formed the higher VM group (EB-HVM; n = 6), whereas those obtained lower than 40% formed the lower VM group (EB-LVM; n = 5). Repeated measures ANOVA on the participants’ sound localization performance only revealed significant Training effect [F_1,20_ = 180.385, *P* < 0.001], but non-significant Group (F_3,20_ = 1.419, *P* = 0.267) and marginally significant Group × Training effect [F_3,20_ = 2.838, *P* = 0.064]. With a corrected threshold (FDR *P* < 0.05), longitudinal analysis showed no significant results in both EB-LVM and EB-HVM groups. The PPI results found significant enhanced functional connectivity between the left precuneus and left occipito-parietal regions in the EB-HVM group, and significant enhanced functional connectivity between the bilateral precuneus and parieto-temporo-frontal regions in the EB-LVM group. The ROI analyses based on the four-group model (all subgroups of EB and LB) revealed only marginally significant Group × Training effect on BOLD signal changes in the left precuneus (F_3,21_ = 2.556, *P* = 0.084, *ƞ* ^*2*^ = 0.277).

To further investigate the role of visuo-spatial working memory on sound localization learning, we conducted correlation analyses between the participants’ performances on the matrix test and improvement on sound localization performance for the EB and LB participants. Significant correlations were revealed for the LB (2D matrix, r = 0.605, *P* = 0.028; 3D matrix, r = 0.544, *P* = 0.055), but not the EB (2D matrix, r = 0.098, *P* = 0.774; 3D matrix, r = 0.177, *P* = 0.602). To exclude the possibility that the significant results are driven by the participants’ learning rather than visual experience, the EB and LB participants were pooled and then divided into the better learner (n = 12, EB/LB = 6/6, improvement > 8%) and poorer learner groups (n = 12, EB/LB = 5/7, improvement ≤ 8%). The behavioral results, as expected, revealed significant Training [F_1,22_ = 367.559, *P* < 0.001] and Group × Training effect [F_1,22_ = 33.796, *P* < 0.001], and a non-significant Group effect [F_1,22_ = 3.037, *P* = 0.095, *ƞ* ^*2*^ = 0.121]. With a corrected threshold (FDR *P* < 0.05), longitudinal analysis only found significant decreases in BOLD responses in the right IPL, right precentral gyrus, left insula, and right STG in the better learner group. The PPI and ROI analyses did not reveal significant results for the better versus poorer learner subgroup method.

## Discussion

There are three main findings in this study. First, visual experience appears to modulate sound localization learning among the LB but not EB group. This effect is indeed unique to the LB as we found normal visual individuals despite their intact visual experience did not gain from the sound localization learning. The precuneus was found to play significantly different roles in the EB and LB groups. The increased activation of the bilateral precuneus after training was correlated with the LB participants’ performance in the sound localization task. By contrast, the EB participants showed decreased activation of the precuneus. These results suggest that the effect of the modulation of the precuneus on sound localization is likely attributable to the prior visual experience encoded by the LB but not the EB participants. This speculation is further substantiated by the different results obtained for the LB-HVM and LB-LVM groups. The higher visuospatial working-memory ability of participants in the LB-HVM group could have modulated the neural processes and hence the sound localization performance. These observations were robust as repeating analyses on other ways of sub-dividing the participants (i.e. EB-HVM and EB-LVM, and poorer/better learners) revealed non-significant or less meaningful results. Second, the role of the precuneus seems to depend on the functionality of the visual system and on prior visual experience. The discovery of enhanced connectivity revealed two distinct neural networks related to the precuneus. Among LB-HVM participants, it is the precuneus-lingual gyrus connectivity, suggesting that sound localization in this group may tap into visuospatial working memory. Among EB participants, it is the precuneus-limbic-multisensory connectivity, suggesting that sound localization in this group may predominantly rely on retrieval from spatial memory. The difference between these networks offers a plausible explanation for the variations in task performance and cross sound localization learning between the LB-HVM and EB participants.

The precuneus is part of the dorsal parietal cortex (DPC) located in the medial part of the PPC (Cabeza et al. 2008). In this study, the increased activation in LB participants after sound localization training was localized to the left and right posterior precuneus. The PPC belongs to the dorsal visual processing stream. It is functionally related to spatial perception (Ungerleider and Mishkin 1982) and specializes in receiving multisensory inputs for spatial processing and integration (Bremmer 2011). The PPC was also found to combine the spatial information embedded in the visual and auditory modalities (Nardo et al. 2014). TMS studies further confirmed the functional role of PPC in multisensory spatial tasks (Azanón et al. 2010; Bolognini et al. 2009). Because the posterior precuneus is connected to the occipital and parietal cortices, it has been implicated in visuospatial processing (Leichnetz 2001), spatial navigation (Boccia et al. 2014), and the retrieval of remembered episodes (Bergström et al. 2013). In this study, the participants learned to relate 15 “Bat-ears” sounds with locations to a specific distance × azimuth during sound localization training. The “Bat-ears” sounds employed for sound localization during the functional scans were recorded at neighboring locations and hence not previously presented to the participants. The successful localization of the sounds would require the participants to decode the embedded spatial information and to generalize the learned sound-to-location relationship to the unlearned sounds. The increased activation of the posterior precuneus in the post-training scan suggested that the LB participants employed active visuospatial navigation processes in sound localization learning. Previous studies of LB individuals focused on neural substrates in the occipital but not parietal cortex (Collignon et al. 2013; Voss et al. 2006) and therefore did not assay the precuneus. Our findings concur with those reported in Voss et al. (2008), which found an increased activation of the precuneus based on comparisons between LB and normal-vision groups.

One interesting finding in this study is that sound localization learning by the LB group was augmented by higher visuospatial working memory ability, which was the case for the LB-HVM but not the LB-LVM group. This was further substantiated by the training-induced enhancement of connectivity between the precuneus and the lingual gyrus in the LB-HVM but not the LB-LVM groups. Such connectivity is likely to be enhanced by prior visual experience and hence by the development of visuospatial working memory in the LB-HVM participants. The existence of the precuneus-lingual gyrus network for mediating sound localization after sound localization learning in the LB-HVM group but not in the LB-LVM group is a new finding. This is consistent with and extends previous findings that the lingual gyrus was associated with the processing of spatial information embedded in sounds in EB (Collignon et al. 2011; Gougoux et al. 2005) and LB individuals (Voss et al. 2006). The precuneus and lingual gyrus were found to be the neural correlates of landmark and path encoding during real-world route learning in people with normal vision (Schinazi and Epstein 2010), which corroborates the involvement of the precuneus-lingual gyrus network during navigation reported in a recent meta-analytic study (Boccia et al. 2014). The identification of the precuneus-lingual gyrus network supports the notion that sound localization learning by the LB-HVM might involve the integration of encoded prior visual experience with the spatial information embedded in the sounds. It is likely to be mediated by visuospatial working memory, which might have developed among the LB-HVM participants who had intact vision in their earlier years of life. Visuospatial working memory was found to augment sound localization learning in those who possessed higher abilities (i.e., LB-HVM individuals) and those who showed significantly higher performance on the sound localization task. Nevertheless, our results did not reveal significant relationships between the years of visual experience and the performance on the matrix tests (measure of visuospatial working memory) in the LB group, which is consistent with the results reported in Cattaneo et al. (2007). Vision was previously found to influence an individual’s development of spatial working memory and spatial imagery (Afonso et al. 2010; Cattaneo et al. 2008; Iachini and Ruggiero 2010). It is highly plausible that the LB participants would have gained very different types of visual exposure from the environment, such as at home, at school, and in the community. After blindness, the differences in prior visual experience would have enabled the LB participants to use different compensatory strategies and to engage in various modes of cross-modal learning. Without a significant relationship with the years of visual experience, our results can support only the notion that, among the LB participants who had higher visuospatial working-memory ability, prior visual experience appears to enhance the activation of the precuneus-lingual gyrus network in sound localization learning. However, the effects of prior visual experience did not seem to be significant for the LB participants, who had lower visuospatial working-memory ability. The extent to which visual experience and exposure to the environment contribute to lower visuospatial working-memory ability among LB individuals calls for future studies. Future study should also include a group of normal vision individuals who receive training on sound localization with comparable task performance with LB individuals. The comparisons between the LB and normal vision individuals will further shed light on the relationships among visual experience, visuospatial working-memory ability and the precuneus-lingual network.

Collignon et al. (2011) revealed that the precuneus-lingual gyrus network in the LB-HVM individuals was different from the lingual gyrus network reported in EB individuals. They found enhanced connectivity between the lingual gyrus and the IPL in congenitally blind participants during sound localization processing. The lack of a normal vision control group in this study cannot provide further evidence to explain our observation that the visuospatial working memory in the LB-HVB participants would have been gained from their prior visual experience. As a result, the notion that prior experience modulates sound localization learning in LB is a speculation that needs to be studied in future research.

Studies of EB individuals have received much more attention than those of LB individuals. Nevertheless, previous studies have often compared EB participants to those with normal vision. The current study revealed decreased activations in the precuneus after the EB participants received sound localization training. It assigns an important role for precuneus in differentiation process underlying sound localization learning between EB and LB individuals. First, the results obtained from the EB cohort were opposite those obtained from the LB, who exhibited increased activity in the precuneus. Second, the left precuneus in the EB group showed enhanced connectivity with an extended network, including the limbic system (the hippocampus and the cingulate gyrus) and the multisensory regions (MTG, STG, IPL). The network was different from that of the LB-HVM group, for whom enhanced connectivity was specific to the lingual gyrus. The decreased involvement of the precuneus among members of the EB cohort is not consistent with the activity reported in other studies. The main reason is that those studies focused on cross-modal spatial processing rather than on cross-modal learning. For instance, both EB and normal vision subjects presented similar involvement of the precuneus during spatial processing (Collignon et al. 2013) and tactile-spatial navigation (Gagnon et al. 2012). Similarly, the precuneus has been reported to be involved during monaural (Voss et al. 2008) and binaural sound localization among EB participants (Gougoux et al. 2005; Tao et al. 2015). In fact, in our study, the level of precuneus activation during the sound localization task was comparable in the LB and EB participants prior to sound localization training. The different patterns of precuneus activation change were observed only after the training. The decreased involvement of the precuneus and its connections with the limbic-multisensory network suggests that the EB participants tended to rely on memory, particularly on the retrieval process, in sound localization learning. Previous studies indicated that the medial and lateral parietal cortices as well as the posterior cingulate cortices are activated in association with memory retrieval (Wartman and Holahan 2013; Weible 2013). This is in agreement with current reports of decreased activity in the precuneus, cingulate gyrus, ITG, MTG, and MOG. The widely distributed precuneus network largely overlaps with the cued memory retrieval network (Burianová et al. 2012). Other studies indicated that the DPC, which contains the precuneus, is associated with the allocation of attention to strategic memory search (Cabeza et al. 2008; Ciaramelli et al. 2008). The left hippocampus has been shown to play a role in memory retrieval (Cabeza et al. 2004), particularly the retrieval of spatial information (DeMaster et al. 2013; Suthana et al. 2011). This is consistent with the finding that the learning of “Bat-ears” sounds by EB participants primarily involves the hippocampus-mediated binding of sound and distance (Chan et al. 2013). Similarly, the concurrent activation of the posterior precuneus and posterior cingulate gyrus was associated with retrieval effects (Elman et al. 2013). The MTG has been reported to mediate the retrieval of semantic (Martin and Chao 2001) and multimodal representations (Visser et al. 2012). Together, these data suggest that the major neural process associated with sound localization learning in the EB participants might be the top-down memory retrieval of the sound-to-distance relationships learned during training (Murray and Ranganath 2007). The localization of new sounds would therefore rely on associating incoming sounds with the sound-location pairs retrieved from memory and the subsequent approximation of the location of the new sound. This process could be quite different from that which the LB participants used—namely, active visuospatial navigation enhanced by visuospatial working memory.

The precuneus is regarded as the neural substrate of several complex cognitive processes (Margulies et al. 2009), including the processing of visuospatial imagery and memory retrieval (Cavanna and Trimble 2006). Recent reports have revealed two distinct networks related to the precuneus: the right parieto-frontal network and the default-mode network (Utevsky et al. 2014). Our findings suggest that the precuneus plays different roles during sound localization training between EB and LB. Its ability to network with different neural substrates perhaps can explain its different roles. Our findings support the notion that the precuneus plays a central role in highly integrated tasks (Cavanna and Trimble 2006). The involvement of the precuneus was found in blind-folded subjects with normal vision, in addition to blind individuals, during sound-to-distance (Chan et al. 2013), tactile-form (Ptito et al. 2012), and spatial navigation learning tasks (Kupers et al. 2010).

This study has several limitations. First, the findings of this study were generated from using a high-resolution sound localization task. This task was meant to tap participants’ ability and performance of sound localization. As a result, the results may not be directly comparable with those obtained by low-resolution sound localization tasks, such as the sound differentiation task (Collignon et al. 2013; Voss et al. 2006). Second, this study did not recruit normal-vision individuals to form a control group. Despite the 7-day training, normal-vision individuals were found to have failed to achieve a higher-than-chance level performance. This did not allow us to verify the observation that visuospatial working memory modulates sound localization learning among LB individuals and those with normal vision. Future studies each should include a normal-vision control group for testing the robustness of this phenomenon. Third, this study subdivided LB participants according to their visuospatial working-memory ability, which resulted in a small sample size for the two subgroups. This compromised the statistical power of the analysis of brain activation and the behavioral performance data. Future study will replicate these experiments with a larger sample size for the LB group.

The current findings shed light on how prior visual experience would modulate sound localization learning by individuals with the early and late onset of blindness. The precuneus appears to play important but different roles in the learning of locating high-resolution sounds among LB and EB individuals. The learning processes undergone among the LB participants are likely to be modulated by their visuospatial working memory, which would have developed prior to the onset of blindness. These learning processes were found to be associated with an enhanced precuneus-lingual gyrus network, suggesting the transformation of auditory information embedded in the stimuli to multisensory spatial representations. In contrast, the EB participants who possessed poor visuospatial working memory appeared to learn the locating of the sounds by encoding and associating the auditory and spatial information embedded in the stimuli without transformation. This binding process was reflected in the memory and precuneus-limbic-multisensory networks revealed in the study. The implication of these findings is that the differences in the learning of sound localization between LB and EB individuals are likely to be attributable to the poor development of visuospatial working memory among the latter. Future research should explore ways in which to improve visuospatial working memory among EB and LB individuals. The outcome is to enable individuals with blindness to better use auditory information for making spatial decisions, such as navigation with or without assistive devices.

## Electronic supplementary material

Below is the link to the electronic supplementary material.

Supplementary material 1 (DOCX 64 KB)
