# Supplementary material for: Prior Visual Experience Modulates Learning of Sound Localization Among Blind Individuals
Source: Brain Topogr. 2017 Feb 4;30(3):364–79. doi: 10.1007/s10548-017-0549-z (PMC5408050; doi:10.1007/s10548-017-0549-z)
Supplement: Supplementary file 1 — Supplementary material 1 (DOCX 64 KB) [file 10548_2017_549_MOESM1_ESM.docx]

Prior Visual Experience Modulates Learning of Sound Localization Among Blind Individuals

Qian Tao ^1,2^

Chetwyn C H Chan ^2*^

Yue-jia Luo ^3^

Jian-jun Li ^4^

Kin-hung Ting ^2^

Zhong-lin Lu ^5^

Susan Whitfield-Gabrieli ^6^

Jun Wang ^3^

Tatia M C Lee ^7,8,9*^

^1^Psychology Department, School of Medicine, Jinan University, Guangzhou, China

^2^Applied Cognitive Neuroscience Laboratory, Department of Rehabilitation Sciences, The Hong Kong Polytechnic University, Hong Kong

^3^National Key Laboratory of Cognitive Neuroscience and Learning, Beijing Normal University, Beijing, China

^4^China Rehabilitation Research Center, Beijing, China

^5^Center for Cognitive and Behavioral Brain Imaging, Arts, & Sciences, Department of Psychology, the Ohio State University, Ohio, OH 43210

^6^The Gabrieli Lab, Massachusetts Institute of Technology, Cambridge, MA 02139 ^7^Laboratory of Neuropsychology, The University of Hong Kong, Hong Kong

^8^Laboratory of Cognitive Affective Neuroscience, The University of Hong Kong, Hong Kong

^9^State Key Laboratory of Brain and Cognitive Science, The University of Hong Kong, Hong Kong

Co-Corresponding Authors:

Chetwyn Chan PhD

Applied Cognitive Neuroscience Laboratory

Department of Rehabilitation Sciences

The Hong Kong Polytechnic University

Hong Kong, China

Tel: 852-2766-6727

E-mail: Chetwyn.Chan@polyu.edu.hk

Tatia M C Lee PhD

Laboratory of Neuropsychology

Department of Psychology

The University of Hong Kong

Hong Kong, China

Tel: 852-3917-8394

E-mail: tmclee@hku.hk

Keywords: Blindness; experience modulation; fMRI; functional connectivity; plasticity; sound localization

Running Head: Experience Modulation on Learning of Sound Localization

**Supplementary Material**

**Table 1**

A summary of significant BOLD responses for the contrast of (Localization – Differentiation) in the EB and LB groups on the pre- and post-training occasions. The thresholds were *P* < 0.001 (uncorrected) at the voxel level and *P* < 0.05 (FWE corrected) at the peak level.

| *x,y,z* (mm) | L/R | Label | Cluster size | *T* | *Z* |
| --- | --- | --- | --- | --- | --- |
| Pre-training fMRI session in EB | | | | | |
| -35,-35,62 | L | Postcentral gyrus | 1603 | 15.89 | 5.61 |
| 34,-22,65 | R | Precentral gyrus | 633 | 11.77 | 5.09 |
| -7,-78,-12 | L | Cuneus | 48 | 5.57 | 3.68 |
|  |  |  |  |  |  |
| Post-training fMRI session in EB | | | | | |
| -38,-22,65 | L | Precentral gyrus | 534 | 11.95 | 5.12 |
| 40,-19,62 | R | Precentral gyrus | 158 | 10.19 | 4.83 |
| -4,-69,49 | L | Precuneus | 177 | 8.40 | 4.47 |
|  |  |  |  |  |  |
| Pre-training fMRI session in LB | | | | | |
| -7,-69,56 | L | Superior parietal lobule | 275 | 9.47 | 4.98 |
| -35,-31,56 | L | Precentral gyrus | 273 | 8.12 | 4.66 |
| -28,-4,56 | R | Middle frontal gyrus | 359 | 7.81 | 4.57 |
|  |  |  |  |  |  |
| Post-training fMRI session in LB | | | | | |
| -7,-66,56 | L | Precuneus | 696 | 11.38 | 5.35 |
| 43,-35,62 | R | Postcentral gyrus | 243 | 6.46 | 4.16 |
| -28,9,65 | L | Middle frontal gyrus | 78 | 5.81 | 3.93 |

**Table 2**

Summary of PPI results performed on the contrast of [(Localization_post_ – Discrimination_post_) – (Localization_pre_ – Discrimination_pre_)] in the EB and LB-HVM groups. *P* < 0.001 (uncorrected) at the voxel level.

| Seed area for PPI analyses | x | y | z | *T* | *P* |
| --- | --- | --- | --- | --- | --- |
| L Precuneus in EB |  |  |  |  |  |
| R Posterior cingulate gyrus | 9 | -66 | 14 | 9.26* | 0.000 |
| L Middle temporal gyrus | -47 | -56 | 1 | 6.72 | 0.003 |
| R Cerebellum | 9 | -47 | -40 | 6.56 | 0.003 |
| L Middle frontal gyrus | -28 | 3 | 46 | 6.26 | 0.007 |
| R Inferior parietal lobule | 34 | -47 | 49 | 5.47 | 0.012 |
| R Middle temporal gyrus | 58 | -0 | -15 | 5.42 | 0.006 |
| R Calcarine gyrus | 21 | -56 | 4 | 5.42 | 0.011 |
| L Cerebellum | -28 | -78 | -31 | 5.22 | 0.007 |
| L Precentral gyrus | -35 | -13 | 43 | 5.22 | 0.009 |
| L Precuneus | -4 | -59 | 52 | 4.95 | 0.015 |
| L Hippocampus | -19 | -35 | -2 | 4.79 | 0.014 |
| R Superior temporal gyrus | 55 | -38 | 14 | 4.59 | 0.015 |
| L Precuneus | -13 | -72 | 33 | 4.55 | 0.015 |
|  |  |  |  |  |  |
| R Precuneus in EB |  |  |  |  |  |
| R Superior temporal gyrus | 58 | -41 | 14 | 5.38 | 0.013 |
|  |  |  |  |  |  |
| L Precuneus in LB-HVM |  |  |  |  |  |
| L Middle occipital gyrus | -35 | -72 | -12 | 11.80 | 0.002 |
| L Precentral gyrus | -35 | -25 | 65 | 10.84 | 0.003 |
| R Lingual gyrus | 9 | -75 | -8 | 10.75* | 0.000 |
| R Cerebellum | 6 | -78 | -40 | 9.33 | 0.001 |
|  |  |  |  |  |  |
| R Precuneus in LB-HVM |  |  |  |  |  |
| L Lingual gyrus | -0 | -87 | -8 | 12.54* | 0.001 |
| L Precentral gyrus | -35 | -28 | 62 | 8.91 | 0.007 |

Significant brain activations after correction over the entire volume (*) or over small volume of interest (SVC). L, left; R, right.
